# Co-ingestion of carbohydrate with leucine-enriched essential amino acids does not augment acute postexercise muscle protein synthesis in a strenuous exercise-induced hypoinsulinemic state

**DOI:** 10.1186/s40064-016-2736-x

**Published:** 2016-08-09

**Authors:** Hiroyuki Kato, Hiromi Suzuki, Yoshiko Inoue, Tetsuya Takimoto, Katsuya Suzuki, Hisamine Kobayashi

**Affiliations:** Frontier Research Laboratories, Institute for Innovation, Ajinomoto Co., Inc, Kawasaki, Kanagawa Japan

**Keywords:** Strenuous exercise, Insulin, Muscle protein synthesis, Leucine-enriched essential amino acids, Hypoinsulinemia

## Abstract

Strenuous exercise following overnight fasting increases fat oxidation during exercise, which can modulate training adaptation. However, such exercise induces muscle protein catabolism by decreasing blood insulin concentrations and increasing amino acid oxidation during the exercise. Leucine-enriched essential amino acids (LEAAs) enhance muscle protein synthesis (MPS) at rest and after exercise. However, it remains to be clarified if the co-ingestion of carbohydrate with LEAAs induces an additional increase in MPS, particularly in a hypoinsulinemic state induced by strenuous exercise. Eight-week-old male Sprague–Dawley rats were made to perform strenuous jump exercise (height 35 cm, 200 jumps, 3-s intervals), after which they ingested distilled water and 1 g/kg LEAAs with or without 1 g/kg of glucose. The fractional synthesis rate was determined by measuring the incorporation of l-[ring-^2^H_5_]-phenylalanine into skeletal muscle protein. Immediately after the exercise, plasma insulin concentration was significantly lower than that at the basal level. Co-ingestion of glucose with LEAAs alleviated the reduction in plasma insulin concentration, while LEAA ingestion alone did not. LEAA administration with or without glucose led to a higher MPS compared with water administration (P < 0.05). However, the co-ingestion of glucose with LEAAs did not induce further increases in MPS compared with LEAA ingestion alone. Thus, the co-ingestion of glucose with LEAAs does not additionally increase MPS under a strenuous exercise–induced hypoinsulinemic state when glucose is co-ingested with a dose of LEAAs that maximally stimulates MPS.

## Background

Skeletal muscles are plastic tissues, which change their phenotype in response to stimuli such as exercise and nutritional availability (Coffey and Hawley 2007). Furthermore, the interaction between training-induced adaptation and nutrient availability has been investigated in detail (Hawley et al. 2011). Classically, high carbohydrate availability has been reported to ensure recovery from endurance exercise (Hawley et al. 1997). However, recent studies have reported that low carbohydrate availability can modify training adaptation (Hawley and Burke 2010). Reduced carbohydrate availability because of low carbohydrate intake or overnight fasting increases fat oxidation during exercise and mitochondrial biogenesis (Hawley and Burke 2010). Thus, endurance athletes should incorporate their training with low or high carbohydrate intake according to their training schedule (Stellingwerf 2012). However, there are some concerns regarding exercise with low glycogen availability. Exercise with low muscle glycogen can enhance amino acid oxidation during exercise (Howarth et al. 2010). Furthermore, a low-carbohydrate diet or overnight fasting before exercise has been associated with a decrease in plasma insulin, a well-known anabolic hormone (Galbo et al. 1979; Weltan et al. 1998). These changes can lead to muscle protein catabolism. Therefore, during the low-carbohydrate training period, close attention should be paid to maintain muscle mass.

The mass of skeletal muscle is maintained by the protein net balance between muscle protein synthesis (MPS) and muscle protein breakdown (MPB). It is well known that resistance exercise alone or that followed by the ingestion of essential amino acids (EAA), leucine-enriched essential amino acids (LEAAs), or protein with or without carbohydrate (CHO) increases MPS in humans (Biolo et al. 1997; Dreyer et al. 2008; Fujita et al. 2007; Rasmussen et al. 2000). Furthermore, protein or amino acid ingestion increases muscle mass during training periods (Cermak et al. 2012). Recently, the importance of protein or amino acid ingestion following endurance exercise has been attracting attention (Moore et al. 2014). In particular, a mixture of LEAAs has been found to induce greater MPS than a standard EAA mixture (Pasiakos et al. 2011). Therefore, the importance of LEAA ingestion following both resistance exercise and endurance exercise is well-accepted.

Although a recent review suggested that CHO should be consumed with protein to maximize muscle hypertrophy by inducing an additive effect of insulin and leucine on protein synthesis (Stark et al. 2012), the necessity of CHO co-ingestion with protein or amino acids to augment postexercise MPS remains unclear (Figueiredo and Cameron-Smith 2013). In fact, hyperinsulinemia is reported to stimulate MPS rates (Biolo et al. 1995; Gelfand and Barrett 1987). However, recent reports suggested that physiological hyperinsulinemia stimulated by the co-ingestion of CHO with protein or amino acid does not induce further increase in MPS (Glynn et al. 2010, 2013; Koopman et al. 2007; Staples et al. 2011). Even basal levels of insulin after fasting are sufficient to enable amino acids to increase MPS under conditions where ample protein is ingested (Greenhaff et al. 2008). On the other hand, insulin secretion is inhibited to below basal levels by adrenergic receptor activation, both via the sympathetic innervation of the islets and by circulating catecholamines (Marliss and Vranic 2002). As a result, insulin concentrations decrease to less than the basal level according to the intensity and duration of the exercise and the duration of fasting before exercise (Vranic et al. 1976). However, whether the co-ingestion of CHO with amino acids can affect the augmented protein synthesis in a hypoinsulinemic state warrants clarification.

Thus, the purpose of this study was to investigate the effect of the co-ingestion of CHO with LEAAs on muscle protein synthesis in a hypoinsulinemic state induced by strenuous exercise following starvation. To this end, we assessed MPS by measuring the fractional synthesis rate (FSR) using the flooding dose method after the ingestion of LEAAs with or without glucose after jumping exercise in overnight fasted rats.

## Methods

### Animals

Eight-week-old male Sprague–Dawley rats (Charles River Laboratories Japan, Inc., Yokohama, Japan) were used in this study after 1 week of habituation. The rats were housed in a temperature-controlled room under a 12-h light–dark cycle. They were also provided standard commercial chow (CR-F1; Charles River Laboratories Japan, Inc.), and water was provided ad libitum throughout the experiment.

### Experimental design

The first step (Experiment 1) was to establish the exercise intensity of jumping exercise by measuring blood lactate concentration during the exercise. Six rats were made to perform the jumping exercise mentioned below after overnight fasting. Before the exercise, after 50, 100, and 200 jumps, blood samples were withdrawn from the tail vein. Immediately after blood sampling, blood glucose and lactate concentrations were measured using the Lactate Pro test meter (Arkray, Kyoto, Japan) and the Dia-sensor blood glucose tester (Arkray).

**Fig. 1:**
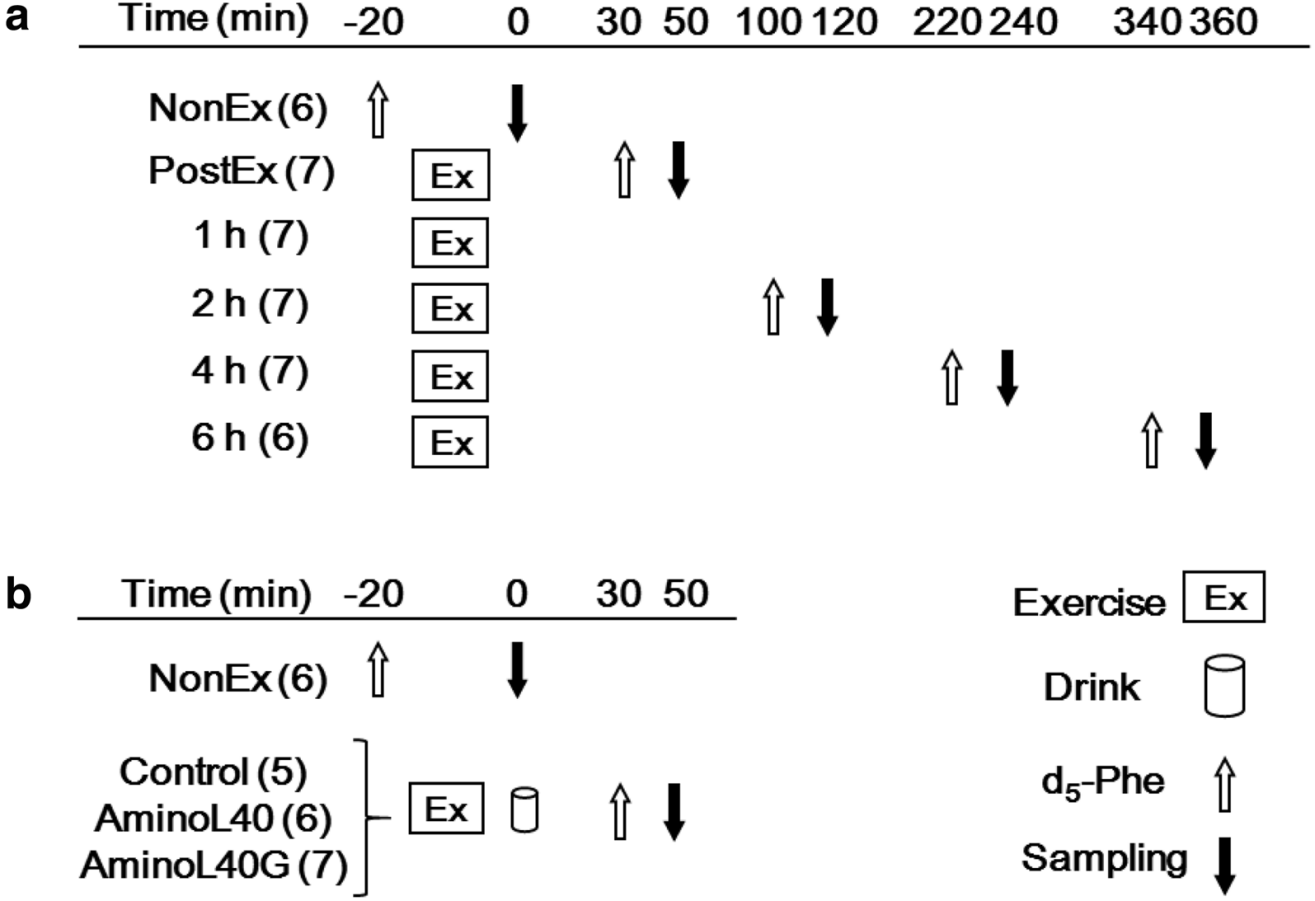
Schematics of the study protocols. **a** Study design of Experiment 2 to measure muscle protein synthesis (MPS) and plasma glucose and insulin concentrations after the jumping exercise following overnight fasting; **b** study design of Experiment 3 to measure MPS and plasma glucose and insulin concentrations after the administration of water (as a control), LEAAs, or LEAAs with glucose after the jumping exercise. The numbers of rats in each group are shown in *parentheses*

Having confirmed the exercise intensity, we proceeded to Experiment 2, in which the effect of the strenuous jumping exercise on plasma insulin and muscle protein synthesis was investigated. The study protocol is shown in Fig. 1a. Forty rats were divided into the following 6 groups: sedentary (NonEx, n = 6); immediately after exercise (PostEx, n = 7); and 1, 2, 4, or 6 h after exercise (n = 7 for 1, 2, and 4 h, n = 6 for 6 h). After overnight fasting, the rats underwent 200 repetitions of jumping exercise. Skeletal muscle protein synthesis was determined as the FSR (%/h) using the flooding dose method as described by Garlick and McNurlan (Garlick and McNurlan 1998). Briefly, rats were injected with flooding doses of phenylalanine (1.5 mmol/kg) containing l-[ring-^2^H_5_]-phenylalanine (50 MPE; Cambridge isotope, Cambridge, MA, USA) intravenously into the tail vein at rest (NonEx); before the exercise (PostEx); and 30 min (1 h), 100 min (2 h), 220 min (4 h), or 340 min (6 h) after the completion of the exercise. Twenty minutes after the tracer injection, blood samples were collected from the abdominal aorta under inhalation anesthesia with 1.5 % isoflurane. The gastrocnemius (GAS) muscle was then removed, frozen in liquid nitrogen, and stored at −80 °C.

Finally, having established the changes in plasma insulin concentrations and MPS after exercise, we proceeded to Experiment 3, in which we investigated the effect of LEAA administration and the addition of CHO to LEAA on MPS at a hypoinsulinemic state induced by strenuous jumping exercise. The study protocol is shown in Fig. 1b. Twenty-four rats were divided into the following 4 groups: sedentary (NonEx, n = 6) and rats administered distilled water as a negative control (Control, n = 5), LEAA mixture (AminoL40, n = 6), or LEAAs with glucose (AminoL40G, n = 7) following jumping exercise. After overnight fasting, rats in the Control, AminoL40, and AminoL40G groups performed the jumping exercise. Immediately after the exercise, rats of the AminoL40 and AminoL40G groups were administered LEAAs (1 g/kg body weight) and LEAAs along with glucose (1 g/kg body weight) by oral gavage, respectively. As controls, rats of the NonEx and Control groups were administered distilled water. Thirty minutes after the oral administration, rats in all the groups were injected with tracer. Twenty minutes after the tracer injection, blood samples were collected from the abdominal aorta, and the GAS muscle was removed under anesthesia.

### LEAAs and glucose

The LEAA mixture consisted of EAAs in the following proportion: histidine, 2 %; isoleucine, 11 %; leucine, 40 %; lysine, 17 %; methionine, 3 %; phenylalanine, 7 %; threonine, 9 %; tryptophan, 1 %; and valine, 11 %. Except for the higher proportion of leucine, this mixture contains the ratio of EAAs found in whey protein. All amino acids were manufactured by Ajinomoto Co., Inc. The AminoL40 mixture was developed with the specific purpose of avoiding a substantial decrease in the availability of the other EAAs while increasing the proportion of leucine. In addition, the AminoL40 mixture has been reported to alleviate MPS after eccentric contraction in rats (Kato et al. 2015). For rescuing the decreased insulin concentration after exercise, 1 g glucose/kg was provided. This dose of glucose was selected to ensure the increase in glucose and insulin concentration after exercise in rats (Anthony et al. 1999).

### Jumping exercise

Rats were made to perform strenuous jumping exercise (height 35 cm, 200 jumps, 3-s intervals) as previously described in detail (Umemura et al. 1997). Such jumping training has been reported to induce an increase in the ratio of type II fiber to type I fiber (Pousson et al. 1991) and bone mass (Umemura et al. 1997). This exercise model was selected to provide strenuous exercise with little acclimatization. Two days before the experiment, the rats were acclimatized to the jumping exercise as follows. The rats were placed in the jumping box, at the bottom of which an electrode plate was installed. Initially, the rats jumped upon electrical stimulation. Through acclimatization, the rats became accustomed to jump without electrical stimulation. On the experimental day, the rats were placed in the jumping box following overnight fasting. The rats then jumped and grasped the top of the box with their forelimbs, after which the rats climbed onto the wall of the box. Subsequently, the rats were caught by the investigators and returned to the bottom of the box for the next jump. This was repeated 200 times, and the total exercise time was roughly 14 min.

### Measurements of blood variables

Blood was separated from plasma by centrifugation at 10,000×*g* for 10 min at 4 °C, and the plasma was stored at −80 °C. Plasma insulin concentrations were measured using a commercial ELISA kit (Morinaga Institute Biological Science, Yokohama, Japan). Plasma amino acid concentrations were measured with an automatic amino acid analyzer (JLC-500; JEOL, Tokyo, Japan). Plasma glucose concentration was assayed for glucose content using the Glucose CII Test Wako kit (Wako Pure Chemical Industries, Ltd., Osaka, Japan) using glucose oxidase.

### Measurement of the FSR

Muscle samples were ground, and intracellular free amino acids and muscle proteins were extracted as previously described (Kato et al. 2015). Subsequently, phenylalanine enrichment (E(muscle free)) in the supernatant was determined by its *tert*-butyl dimethylsilyl derivatization (N-methyl-N-*tert*-butyldimethylsilytrifluoroacetamide; Thermo Fisher Scientific, Waltham, MA, USA) using gas chromatography–mass spectrometry (GC–MS; 6890 GC system and 5973 Network Mass Selective Detector, Agilent, Santa Clara, CA, USA) to monitor ions 336 and 341 in the electron impact mode. Muscle protein-bound phenylalanine enrichment [E(protein-bound)] was determined by measuring the butyl derivatization (HCl-*n*-butanol [10 v/v %]: GL Science Inc., Tokyo, Japan) using liquid chromatography–mass spectrometry to monitor ions 224 and 227 at the first mass spectrometry, and 122 and 125 at the second mass spectrometry (LC–MS/MS; Prominence HPLC system, Shimadzu, Kyoto, Japan and API 3200, SCIEX, Framingham, MA, USA) using the external standard curve approach (Calder et al. 1992). The FSR of GAS muscle protein was calculated with the precursor-product model as previously described (Kato et al. 2015). Briefly, MPS was calculated as follows: FSR (%/h) = E (protein-bound)/(E (muscle free) × t) × 100, where t represents the time interval between phenylalanine injection and tissue sampling.

### Statistical analysis

Values are shown as mean ± SEM. Repeated-measures ANOVA followed by Bonferroni’s multiple comparison test was used to analyze the changes in blood glucose and lactate concentrations in Experiment 1. One-way ANOVA followed by Bonferroni’s multiple comparison test was performed to test the changes in the other parameters. All the statistical analyses were performed using GraphPad Prism 5 (GraphPad Software Inc., San Diego, CA, USA). Values of P < 0.05 were considered significant.

## Results

### Blood glucose and lactate concentrations during jumping exercise

Blood glucose was significantly lower after 200 jumps than that before the jumps (Table 1, P < 0.01). Blood lactate concentration increased significantly after 50 jumps and remained high until 200 jumps, compared with the pre value (Table 1, P < 0.01). The intensity of exercise was considered strenuous or high when lactate concentrations were >4 mM.

**Table 1:** Blood glucose and lactate concentrations during dynamic exercise

Variables	Pre value	Jump		
		50	100	200
Glucose, ng/mL	4.5 ± 0.2	4.6 ± 0.5	3.9 ± 0.5	3.5 ± 0.4**
Lactate, mM	1.8 ± 0.1	4.6 ± 0.4**	4.9 ± 0.6**	5.0 ± 0.5**

Data are shown as mean ± SEM (n = 6); ** P < 0.01 (significantly different from the pre value)

### Changes in plasma glucose and insulin concentrations and MPS after jumping exercise

**Fig. 2:**
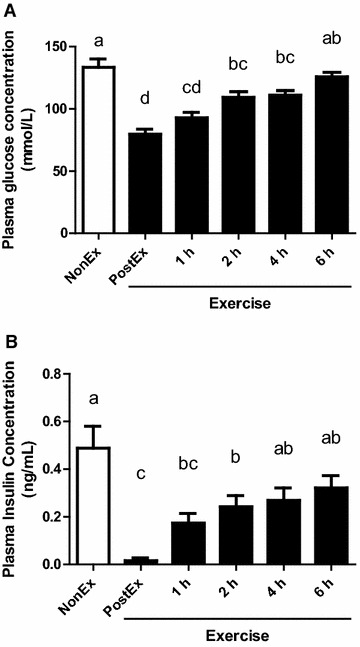
Plasma glucose (**A**) and insulin (**B**) concentrations in sedentary (NonEx; *open bars*) and exercised (ex; *solid bars*) groups of rats studied 1, 2, 4, and 6 h after dynamic exercise. Data are shown as mean ± SEM (n = 6 for the NonEx and 6 h groups, and n = 7 for the PostEx, 1 h, 2 h, and 4 h groups). *Different letters* denote significant difference (P < 0.05)

**Fig. 3:**
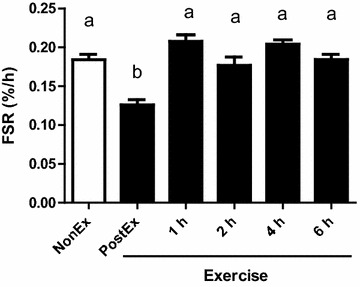
Rates of protein synthesis for mixed gastrocnemius muscle in sedentary (NonEx; *open bars*) and exercised (ex; *solid bars*) groups of rats studied 1, 2, 4, and 6 h after dynamic exercise. Data are shown as mean ± SEM (n = 6 for the NonEx and 6 h groups, and n = 7 for the PostEx, 1 h, 2 h, and 4 h groups). *Different letters* denote significant difference (P < 0.05)

The plasma glucose concentration significantly decreased immediately after the jumping exercise (PostEx), gradually returning to the level of the NonEx groups until 6 h after exercise (Fig. 2A). Accordingly, the plasma insulin concentration decreased significantly immediately after the jumping exercise (PostEx), recovering 4 h after the exercise (Fig. 2B). The FSR in GAS muscle protein was significantly lower in the PostEx group compared with all the other groups (Fig. 3, P < 0.05).

### Changes in MPS after the administration of LEAAs with or without glucose after jumping exercise

**Fig. 4:**
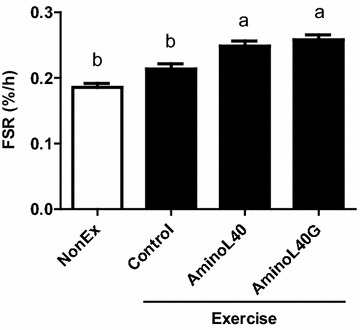
Rates of protein synthesis for mixed gastrocnemius muscle in sedentary (NonEx; *open bars*) and exercised (ex; *solid bars*) groups of rats administered water (Control), AminoL40 (1 g LEAA/kg), or AminoL40G (1 g LEAA + 1 g glucose/kg) after exercise. Data are shown as mean ± SEM (n = 6 for the NonEx and AminoL40 groups, n = 5 for the Control group, and n = 7 for the AminoL40G group). *Different letters* denote significant difference (P < 0.05)

Although the jumping exercise alone did not increase MPS 1 h after the exercise, the administration of LEAAs after the jumping exercise increased MPS compared with that in the NonEx and Control groups (Fig. 4, P < 0.05). The co-ingestion of CHO with LEAAs following the jumping exercise increased MPS, while it did not induce any further increase in MPS compared with that in the AminoL40 group (Fig. 4).

### Blood variables after the administration of LEAAs with or without glucose following jumping exercise

**Fig. 5:**
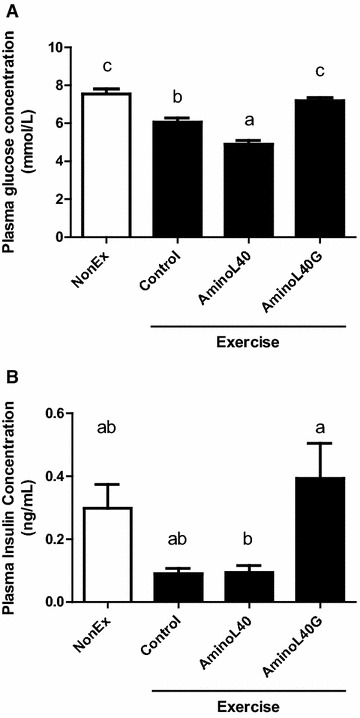
Plasma glucose (**A**) and insulin (**B**) concentrations in sedentary (NonEx; *open bars*) and exercised (ex; *solid bars*) groups of rats administered water (Control), AminoL40 (1 g LEAA/kg), or AminoL40G (1 g LEAA + 1 g glucose/kg) after exercise. Data are shown as mean ± SEM (n = 6 for the NonEx and AminoL40 groups, n = 5 for the Control group, and n = 7 for the AminoL40G group). *Different letters* denote significant difference (P < 0.05)

Plasma glucose concentration was significantly lower in the Control group than that in the NonEx group (Fig. 5A). Moreover, the administration of LEAAs induced a further decrease in plasma glucose concentration compared with those in the NonEx and Control groups. On the other hand, the administration of LEAAs with glucose recovered plasma glucose concentration to the level of the NonEx group (Fig. 5A). The plasma insulin concentration after the administration of LEAAs with glucose was significantly greater than with the administration of LEAAs alone (Fig. 5B, P < 0.05).

Plasma amino acid concentrations are shown in Table 2. EAA concentrations, except for those of His and Trp, were significantly greater (2–9-fold greater) in the AminoL40 group than those in the NonEx and Control groups (Table 2, P < 0.05). Furthermore, the co-ingestion of glucose with LEAAs decreased the concentrations of Ile, Leu, Lys, and Val compared with those in the AminoL40 group, whereas the concentrations of EAAs except for His and Trp were significantly higher in the AminoL40G group compared with the NonEx and Control groups (Table 2, P < 0.05).

**Table 2:** Plasma essential amino acid concentrations in sedentary (NonEx) and exercised groups of rats administered water (Control), LEAA (AminoL40; 1 g LEAA/kg), or LEAA + glucose (AminoL40G; 1 g LEAA + 1 g glucose/kg) after exercise

Amino acid, µmol/L	NonEx	Control	AminoL40	AminoL40G
His	50.8 ± 3.8	48.3 ± 7.3	56.2 ± 4.9	51.8 ± 4.9
Ile	98.1 ± 14.4 a	110.9 ± 14.7 a	443.3 ± 50.8 b	326.3 ± 46.2 c
Leu	149.0 ± 16.0 a	175.8 ± 32.7 a	1594.0 ± 177 b	1219.6 ± 163.6 c
Lys	558.3 ± 79.9 a	566.3 ± 142.7 a	1499.2 ± 170.7 b	1175.7 ± 165.8 c
Met	64.4 ± 13.1 a	83.0 ± 17.4 a	131.2 ± 13.2 b	125.1 ± 15.5 b
Thr	314.5 ± 39.5 a	311.5 ± 61.4 a	435.7 ± 59.5 b	515.0 ± 89.7 b
Trp	94.1 ± 20.1	95.4 ± 13.0	84.1 ± 9.4	98.9 ± 18.1
Val	269.1 ± 30.4 a	328.7 ± 52.5 a	1073.9 ± 125.1 b	869.9 ± 56.1 c

Data are shown as mean ± SEM (n = 6 for the NonEx and AminoL40 groups, n = 5 for the Control group, and n = 7 for the AminoL40G group). Different letters denote significant difference (P < 0.05)

## Discussion

The objective of this study was to investigate the effect of the co-ingestion of glucose with LEAAs on MPS under a hypoinsulinemic state induced by strenuous exercise following overnight fasting. First, by measuring insulin concentration after jumping exercise, we established the hypoinsulinemic state induced by jumping exercise. In addition, MPS was suppressed during exercise, reverting to the level of the sedentary rats 1 h after the exercise. Second, LEAA administration with or without glucose following strenuous exercise augmented MPS. However, the co-ingestion of glucose with LEAAs did not induce any further increase in MPS compared with LEAAs alone, despite a recovery of the decrease in insulin concentration. Leucine-enriched protein feeding was recently reported to not impair exercise-induced fat oxidation during carbohydrate-restricted training (Impey et al. 2015). Therefore, LEAA supplementation without carbohydrate intake is assumed to contribute to maintaining lean body mass without impairing training-induced adaptation during carbohydrate-restricted training.

Although amino acids, particularly leucine, are known to stimulate insulin secretion (Crozier et al. 2005; Glynn et al. 2013; Grasso et al. 1976), LEAA administration alone did not alleviate the decrease in insulin concentration induced by strenuous exercise after overnight fasting. In previous studies, leucine or protein ingestion after exercise induced no or minimal increase in insulin concentration (Anthony et al. 1999; Koopman et al. 2007; Staples et al. 2011). Therefore, the effect of amino acid or protein ingestion on insulin secretion is not sufficient to increase insulin concentration after exercise. In contrast to the ingestion of LEAAs alone, the co-ingestion of glucose with LEAAs reversed the insulin concentration to the basal level. However, the recovery of insulin concentration by adding glucose did not lead to any further increase in MPS, which was augmented by LEAA administration. Our results are consistent with former studies (Koopman et al. 2007; Staples et al. 2011), where hyperinsulinemia did not induce further increase in MPS compared with protein and/or amino acid ingestion. Therefore, based on our present findings and former studies, we surmise that the co-ingestion of CHO with protein or amino acids does not increase MPS, regardless of insulin concentrations. Leucine is also known to enhance protein synthesis by stimulating the mammalian target of rapamycin (mTOR) pathway (Crozier et al. 2005). Moreover, insulin affects mTOR activity by stimulating the insulin receptor substrate-1-Akt pathway (Norton and Layman 2006). Therefore, the lack of insulin mediated-augmentation of MPS reflected the fact that insulin shares the molecular pathway to stimulate MPS with leucine. In the current study, the dose of leucine administered was 0.4 g/kg, which is considered sufficient to maximize MPS (Crozier et al. 2005). However, in a different study, insulin co-ingestion with amino acid increased MPS when the administered dose of amino acid was not sufficient to augment MPS (Fryburg et al. 1995). Therefore, when a smaller amount of LEAA is provided, the additive effect of co-ingested CHO might increase MPS.

In addition to the effect of insulin on MPS, insulin has been reported to inhibit MPB without the ingestion of amino acids (Gelfand and Barrett 1987). In addition, amino acids may enhance this effect (Flakoll et al. 1989). Moreover, hyperinsulinemia has been reported to attenuate MPB following resistance exercise (Borsheim et al. 2004; Roy et al. 1997). Although MPB was not investigated in the current study, lower plasma concentrations of Leu, Ile, Val, and Lys were found after CHO co-ingestion compared to the plasma concentrations of these EAAs after the ingestion of LEAAs alone. This suggests that protein breakdown was reduced. Repeated, acute, net-positive protein balance induced by exercise results in chronic adaptation (i.e. muscle hypertrophy) (Phillips 2014). However, MPB is likely to have a smaller impact on hypertrophy than MPS, because the magnitude of change in MPB is much lower than that in MPS (Glynn et al. 2010).

The MPS response after an acute intervention (nutrition and/or exercise) corresponds to changes in muscle hypertrophy (Burd et al. 2010a, b; Hartman et al. 2007; Mitchell et al. 2012; Wilkinson et al. 2007). Thus, acute measurements of MPS can provide important insight into the mechanism of induction of muscle hypertrophy and/or suppression of muscle atrophy. However, muscle hypertrophy after prolonged resistance training did not show a linear relationship with acute MPS after resistance exercise within the same subjects (Mitchell et al. 2014). Therefore, further studies are required to clarify the long-term effect of LEAAs on lean body mass and training adaptation during carbohydrate-restricted training.

In conclusion, our present results indicated that when a sufficient amount of LEAAs for maximizing MPS is provided, the co-ingestion of glucose with LEAA intake is not necessary to induce a maximal increase in MPS, even under a very low plasma insulin concentration induced by strenuous exercise following overnight fasting. Further studies are required to clarify the long-term effect of LEAAs on the protein metabolism, muscle mass and training adaptation.

## Abbreviations

GAS: gastrocnemius
EAAs: essential amino acids
LEAAs: leucine-enriched essential amino acids
MPS: muscle protein synthesis
MPB: muscle protein breakdown
mTOR: mammalian target of rapamycin
